# A Healthy Diet Rich in Calcium and Vitamin C Is Inversely Associated with Metabolic Syndrome Risk in Korean Adults from the KNHANES 2013–2017

**DOI:** 10.3390/nu13041312

**Published:** 2021-04-16

**Authors:** Sunmin Park, Kyungjin Kim, Byung-Kook Lee, Jaeouk Ahn

**Affiliations:** 1 Department of Food and Nutrition, Obesity/Diabetes Center, Hoseo University, Asan 31499, Korea; smpark@hoseo.edu; 2 Graduate School of Medical Informatics, Soonchunhyang University, Asan 31538, Korea; spinedr@naver.com; 3 Department of Preventive Medicine, Soonchunhyang University, Asan 31538, Korea; bklee@sch.ac.kr

**Keywords:** metabolic syndrome, healthy eating index, Ca, vitamin C, dietary fiber

## Abstract

The association between metabolic syndrome and eating patterns remains unclear. We hypothesized that Korean Healthy Eating Index (KHEI) scores were related to metabolic syndrome (MetS) risk in adults in a gender-dependent manner. We aimed to examine the hypothesis using the Korea National Health and Nutrition Examination Survey-VI (2013–2017) data with a complex sample survey design. Adjusted means and 95% confidence intervals of KHEI scores and nutrient intake estimated by the 24-h recall were calculated according to MetS status after adjusting for age, residence area, region, education, obesity, income, drinking status, smoking status, marriage, and exercise. Adjusted odds ratios for MetS were measured according to KHEI quartiles using logistic regression analysis while controlling for covariates. MetS incidence was significantly higher in females than in males. Those who were older, less educated, earning less income, more obese, living in rural areas, drinking severely, non-exercising, and married had higher MetS incidence than those with the opposite state. Total KHEI scores of all components KHEI scores were lower for those with MetS (MetS group) than those without MetS (Non-MetS group) in both genders. For KHEI components, having breakfast and milk and fat intake had lower scores for the MetS group than for the Non-MetS group in women, whereas fruits and milk and milk product intake had lower scores for the MetS group in men. Nutrient intake influenced the MetS risk in females more than in males. Fat, calcium, and vitamin C intakes from 24-h recall were lower in the MetS group than in the Non-MetS group in women. KHEI scores had an inverse association with MetS risk by 0.98-fold in both genders after adjusting for covariates. In conclusion, a healthy diet that includes adequate calcium and vitamin C is associated with a lower the risk of MetS in both men and women.

## 1. Introduction

Metabolic syndrome (MetS) is a cluster that simultaneously has three out of five abnormal conditions: abdominal obesity, hypertension, hyperglycemia, hypo-HDL-cholesterolemia, and hypertriglyceridemia [1]. Persons with MetS are at a greater risk of type 2 diabetes and cardiovascular diseases [2]. MetS prevalence is rising worldwide. In Asia, it remarkably increased in the last decades due to the Westernization of lifestyles, including food intake and a sedentary lifestyle. Asians have traditionally eaten unrefined grain-based diets with vegetables for a long period. They maintain serum glucose concentration within a normal range with low insulin secretion, suggesting that Asians have a high insulin sensitivity to normalize serum glucose concentration after a high carbohydrate intake, including high dietary fiber. However, their dietary patterns have changed in the last decades. They consume a lot of refined grains. They have a low dietary fiber intake but increased fat intake, although fat intake in Asian countries is still much lower than in Western countries. Koreans’ average fat intake is about 20–25%, although it showed some age differences in 2017 [3]. Such dietary changes have elevated insulin resistance that requires higher insulin secretion to maintain normoglycemia. However, Asians are susceptible to hyperglycemia since they have lower insulin secretion capacity with genetic and environmental impacts [4,5]. Therefore, Asians can quickly develop type 2 diabetes when they have MetS. Optimal dietary interventions for MetS prevention and management need to be investigated. Dietary modification is the primary preventive and therapeutic measure.

Healthy eating index (HEI) reflects a wide variation in the magnitude of diet-quality changes in the USA [6,7]. It can be used in the intervention of cardiometabolic risk conditions in a systematic review [8,9]. The Korea Center for Diseases and Control (KCDC) has designed the Korean Healthy Eating Index (KHEI) for adults by modifying HEI to reflect Korean eating habits to influence metabolic diseases based on previous research, dietary guidelines for Koreans, and the Dietary Reference Intake for Koreans [10]. The central theme for establishing KHEI has been related to nutritional interventions for metabolic syndrome, including obesity, abdominal obesity, dyslipidemia, hypertension, and hyperglycemia. KHEI can be used to assess the diet quality of Korean adults [10]. KHEI comprises three major components: dietary adequacy, moderation of nutrient intake, and a diet balance [2]. The adequacy part includes having breakfast and the intakes of mixed grain, fruits and fruit juice, vegetables, fermented vegetables, meats, and milk and milk products. The moderation part contains the intake of saturated fatty acids, sodium, and sweets and beverage. The balance part includes energy intake [10]. These factors of KHEI are well-established factors that can influence the risk of metabolic diseases in Korean adults. For each item of KHEI, a higher value indicates healthier eating. For Korean adults in the Korea National Health and Nutrition Examination Survey (KNHANES)-VI (2013–2015), the intakes of grains, fish, and meats, milk, and dairy food intake, having breakfast, sodium intake, percentages of energy intake from empty calorie foods, and percentages of energy intake from fat are given a score of 10 each [2]. The rest of the items were given a score of 5 each. Total scores for 14 items of KHEI are 100 points. In Korea, adults’ average scores are 63.3, with men having lower average scores than women. Adults in their 20s and 30s have the lowest scores among all age groups [10]. These results indicate that KHEI scores can properly reflect Korean dietary patterns. Thus, KHEI can be used for monitoring dietary quality for adults.

However, the association of KHEI with metabolic syndrome risk remains unclear. We hypothesized that KHEI was associated with metabolic syndrome and its components in a gender-dependent manner in adults. In the present study, we aimed to examine the hypothesis using the KNHANES-VI (2013–2017).

## 2. Methods

### 2.1. Design and Data Collection

This study analyzed data from KNHANES-VI 2013–2017, which studied 12,317 adults with ages ranging from 20–64 years. Annual KNHANES surveys utilize a rolling sampling design and a complex, stratified, multistage probability-cluster survey to obtain a representative sample of free-living South Korean civilians [1]. The KNHANES is a large representative sample of the population. The Korean Centers for Disease Control and Prevention and the Korean Ministry of Health and Welfare conducted the rigorously controlled survey. Health status was evaluated using both interviews and physical examinations, and a survey was used to evaluate nutritional status. The study was conducted in accordance with the Helsinki Declaration of 1975, 2008 revision, and was approved by the Institutional Review Board of the Korean Centers for Disease Control and Prevention (approval no. 2013-07CON-03-4C).

The present cross-sectional analysis utilized data from subjects between the ages of 20–64 years who completed both the health examination and nutrition surveys (*n* = 12,317). The surveys have been described in detail previously [1]. The health interview recorded age, residence place, education level, income, alcohol and tobacco use, and exercise habits. The health examination measured height and weight (wearing light clothing and no shoes). Body mass index (BMI) was calculated by dividing kg body weight by height in meters squared (kg/m^2^). Asian obesity definitions were used to categorize participants into three body types according to recommendations of the International Obesity Task Force and the World Health Organization (WHO) Regional Office for the Western Pacific Region: lean (BMI < 18.5), normal (18.5 ≤ BMI < 25), and obese (BMI ≥ 25) [11]. Participants were also divided into five age groups using the reported ages at the interviews: (20–29, 30–39, 40–49, 50–59, and 60–64 years). Area of residence was categorized into the following five regions: (1) Seoul, Incheon, Kyunggi area and Gangwon-area, (2) Chunngchung area, Daejeon, and Sejong, (3) Kyungbook, and Daegu, (4) Busan and Kyungnam area, and (5) Jeonbuk and Jeonnam area, Kwangju, and Jeju area [9]. Family Incomes were divided into four quartile groups. Education level was categorized into below high school, high school, and college or higher.

Amounts of alcohol consumed by the participants were estimated by questioning them about average frequency (days per month) on consumption and volumes (mL) and types of alcoholic beverages ingested on a single occasion. The data obtained were used to convert the beverage consumption into the amount of pure alcohol (in grams) consumed per day. The drinking habits of each participant were classified as none, mild (1–15 g), moderate (16–30 g), and heavy (> 30 g), based on previous studies of the influence of alcohol consumption on alcohol-related diseases [12,13]. Participants were considered smokers or non-smokers (having smoked less than 100 cigarettes in a lifetime) or past smokers if they smoked more than 100 cigarettes but not in the last six months [14]. KNHANES defined regular exercise as having ≥30 min of moderate exercise activities at least five times per week or ≥20 min of vigorous exercise activities three or more times per week. The moderate exercise included swimming slowly, playing doubles tennis or volleyball, and participating in occupational or recreational activities while carrying light objects. Running, climbing, cycling fast, swimming fast, playing football, basketball, squash or singles tennis, jumping rope, and participating in occupational or recreational activities while carrying heavy objects were considered vigorous exercises [15].

### 2.2. Definition of Metabolic Syndrome

Criteria from the 2005 revised National Cholesterol Education Program-Adult Treatment Panel III was used to define metabolic syndrome as having three or more of the following [16]: (1) elevated blood pressure (average systolic blood pressure >130 mmHg or diastolic blood pressure >85 mmHg) or current blood pressure medication use; (2) low serum concentrations of high-density lipoprotein (HDL)-cholesterol (<40 mg/dl for men and <50 mg/dL for women); (3) serum triglycerides (TG) level (≥150 mg/dl) or current pharmaceutical treatment of high TG (4) high fasting blood glucose concentrations (≥110 mg/dl) or current anti-diabetic treatment; (5) abdominal obesity (waist circumferences for men and women of ≥102 and 90 cm, respectively).

### 2.3. Laboratory Testing

Overnight fasting blood samples collected in the morning were used to prepare serum. An autoanalyzer (Hitachi Ltd., Tokyo, Japan) was used to determine serum concentrations of glucose, HDL, TG, aspartate transaminase (AST), and alanine transaminase (ALT). The Friedwald equation was used to calculate low-density lipoprotein cholesterol (LDL) as follows: LDL = total cholesterol—HDL—(TG/5), when TG was <400 mg/dL [2,17]. When TG was above 400 mg/dL, TG was measured directly on the Hitachi 7600 auto-analyzer. Neodin Medical Institute, a laboratory certified by the Korean Ministry of Health and Welfare, performed all clinical analyses.

### 2.4. Food and Nutrient Intakes from 24-h Recall and Food Frequency Questionnaire (FFQ)

Participants were directed to continue their regular dietary habits prior to assessing dietary intakes. Daily nutrient intakes were estimated using a 24-h recall questionnaire administered in-person by trained dieticians at mobile examination centers. The data were used to calculate intakes of calories and nutrients on a daily basis using the Can-Pro 2.0 nutrient assessment program developed by the Korean Nutrition Society. 

Dietary intake information was obtained from each participant by using a validated semi-quantitative food frequency questionnaire (SQFFQ) that was designed and validated by the Ministry of Health and Welfare [18]. This questionnaire recorded participant’s estimates of the consumption of 113 food items during the last year. Food intake frequencies were quantified according to the following nine categories: never or seldom, once a month, two to three times a month, one to two times a week, three to four times a week, five to six times a week, once a day, twice a day, and three times or more every day [18].

### 2.5. Korean Healthy Eating Index (KHEI) Scores

KCDC developed KHEI to make comprehensive assessments of Koreans’ diet quality. It included the adequacy of food intake, appropriateness of saturated fat, sugar, and sodium intakes, and balance of energy, fat, and carbohydrate intakes [2,19,20]. KHEI scores were calculated using SQFFQ. KHEI was composed of adequacy (8 items), moderation (3 items), and balance (3 items) domains of energy intake. Their standards for maximum scores of adults aged 20–64 years are given in Supplemental Table S1. Components in the adequacy domain and their top scores were: (1) having breakfast (5–7 times/week), (2) mixed grains intake (≥0.3 serving/day), (3) total fruits intake (≥3 serving/d for men and ≥2 serving/d for women), (4) fresh fruits intake (≥1.5 serving/d for men and ≥1 serving/d for women), (5) total vegetable intake (≥8 serving/d for men and ≥6 serving/d for women), (6) vegetable intake excluding kimchi and pickled vegetable intake (≥5 serving/d for men and ≥3 serving/d for women), (7) meat, fish, eggs, and beans intake (≥5 serving/d for men and ≥2.5 serving/d for women), and (8) milk and milk products intake (≥1 serving/day). Moderation of nutrient intake containing 3 items with their standards for maximum scores of adults aged 19–64 years was provided as follows: (1) saturated fatty acids (≤7% of total energy intake), (2) sodium intake (≤2000 mg/day), and (3) sweets and beverages (≤10% of total energy intake). Balance of energy intake also included three items with their standards for maximum scores of adults aged 19–64 years was provided as follows: (1) energy intake from carbohydrate (55–65% of total energy intake), (2) energy intake from fat (15–30% of total energy intake), and (3) energy intake (75–125% of the estimated energy intake requirement). Each item was given a score, with a possible total score between 0 and 100. Their higher scores indicated having healthier diets. The maximum KHEI score was determined using data from the Dietary Guidelines for Korean Adults and dietary reference intake (DRI) for Koreans in 2010 [21]. However, the KHEI did not include scores for sweets and beverage intake, although their intake should be reduced based on the recommendation of WHO/Food and Agriculture Organization (FAO) [22].

### 2.6. Statistical Analysis

All statistical analyses were conducted using SAS version 9.4 (SAS Institute, Cary, NC, USA) or SUDAAN 11.0 (Research Triangle Institute, Research Triangle Park, NC, USA), which incorporates sample weights and adjusted analyses for analyzing data from complex studies. Weighted data from surveys were used in all analyses to generate representative estimates for the free-living civilian Korean population. *p* values < 0.05 were considered to be statistically significant for all determinations.

According to metabolic syndrome status, descriptive statistics of participants from frequency distributions of categorical demographic variables such as lifestyle factors and metabolic syndrome components were evaluated. Chi-squared tests determined the significances of differences in categorical variables. Adjusted means and 95% CI of KHEI scores and mean intakes of major nutrients analyzed using the Satterthwaite chi-square test according to genders by multiple regression analysis after adjusting for covariates (sex, age, residence area, occupation, income, education level, marriage, drinking status, obesity, and physical activity).

Finally, adjusted odds ratios (ORs) and 95% confidence intervals (CI) for the risk of metabolic syndrome and related conditions according to KHEI quartile scores were calculated using logistic regression after adjusting for sex, age, residence area, occupation, income, education level, marriage, drinking status, obesity, and physical activity.

## 3. Results

### 3.1. General Population Features According to Metabolic Syndrome

Significant differences were observed for age, gender, education, income, smoking and alcohol drinking status, exercise, marital status, and survey years between the groups with and without MetS (Table 1). The MetS incidence was highest in women, those in rural areas, current smokers, and mild and moderate drinkers (Table 1). The elderly, those who were obese, sedentary, unmarried, those who had lower than high school education, and those who had a lower income had the highest incidences of MetS. The incidence of MetS in 2015 and 2016 increased compared to that in 2013 and 2014 in the survey (Table 1).

### 3.2. Distribution of Socioeconomic and Lifestyle Variables of Participants According to Quartiles of KHEI Scores

Women belonged to the lowest quartile (Q1) of KHEI much more than men. However, women were much higher in Q2 and Q3 sections than men. KHEI scores were different among different age groups, with participants aged 30–39 years having the lowest KHEI score and low quartiles than those aged >40 years (Table 2). Younger people aged 20–39 years had worse eating habits than the elderly aged >50 years. Participants having higher than college education were lower in the lowest quartile of KHEI than those with less than high school education (Table 2). Participants having higher income were lower in the lowest KHEI quartile higher in the highest quartile. Regarding smoking status, past smokers had better KHEI than non-smokers. Non-smokers belonged to the lowest quartile of KHEI more than past-smokers and the highest quartile lower than past-smokers and current-smokers (Table 2). Thus, non-smokers had worse eating habits than smokers and past smokers. For drinking status, participants with non- or mild drinking belonged to the lowest quartile of KHEI scores, lower than those with moderate- or severe alcohol drinking (Table 2). Participants of the exercise group belonged to the highest quartile of KHEI scores more than those in the non-exercise group. Married participants belonged to the highest quartiles higher than unmarried persons. Fewer participants belonged to the highest quartile in 2016 than in 2013 (Table 2).

### 3.3. KHEI Scores in Each Gender According to the Presence of Metabolic Syndrome (MetS)

KHEI is composed of adequacy, moderation, and balance domains of energy intake. There was a gender difference in the KHEI score (Table 3). Among the adequacy domain components for food intake, the KHEI score for having breakfast was lower in the MetS group than the Non-MetS group for females but not males. Scores of total fruits and fresh fruits intake were lower in the MetS group of males only (Table 3). Both genders had higher scores for milk and milk product intake Non-MetS group than in the MetS group (Table 3). Scores of the moderation domain for nutrient intake (saturated fatty acids, sodium, and sweets) showed no significant difference between MetS and Non-MetS groups for both genders (Table 3). Among the balance of energy nutrient intakes, energy intake scores from fat were lower in the MetS group than in the Non-MetS group, but only for women. Total scores of KHEI components for both genders were much higher in the Non-MetS group than the MetS group (Table 3).

### 3.4. Macronutrient Intake Estimated by 24-h Recall

Energy intake calculated by 24 h recall was not different according to gender or MetS (Table 4). However, the intake of some nutrients was significantly different between MetS and the Non-MetS groups. These differences partly matched with KHEI scores in MetS incidence (Table 4). Participants with MetS had lower fat and protein intake but higher carbohydrates than those without MetS in women, not in men. However, calcium intake related to milk and its products was significantly lower in the MetS group than the Non-MetS group only in women. Vitamin C intake related to fruit and vegetable intake was significantly lower in the MetS group than in the Non-MetS group for both genders. However, dietary fiber intake was not significantly different between MetS and Non-MetS groups (Table 4).

### 3.5. Association of KHEI Scores with MetS Status

KHEI scores were divided into four groups by quartiles. The lowest quartile (Q1) was used as the reference in logistic regression analysis. Q1 and Q4 indicated the worst and best dietary habits, respectively. KHEI scores had negative associations in both genders after adjusting for different covariates (Table 4). In model 1, adjusted odds ratios for having MetS had negative associations with the Q2, Q3, and Q4 of KHEI by 0.773-, 0.694-, and 0.528-fold, respectively, compared to the Q1 in both genders. There were also negative associations between KHEI scores and MetS risk in both genders in models 2 and 3, adjusting for different covariates known to be related to MetS incidence (Table 5).

## 4. Discussion

MetS is defined as the coexistence of 2–3 metabolic disturbances, including central obesity, hypertension, hyperglycemia, and dyslipidemia, although their cutoffs are somewhat different depending on the organism [1]. In this study, the definition by 2005 revised National Cholesterol Education Program-Adult Treatment Panel III criteria was applied [16]. MetS affects approximately 10–50% of adults, and its prevalence is continuously rising worldwide. The common cause is elevated insulin resistance. Changes in lifestyles and aging are also involved in its increased prevalence. The number of elderly is growing worldwide. Thus, the increase of MetS incidence is inevitable. Changes in lifestyles can prevent it or delay it. Asians have consumed high whole grains and low meats for a long time. However, they had refined grains and more meats in the last three decades due to improved economic status. Decreased dietary fiber intake can result in increased glycemic index, thus elevating the MetS risk. Impacts of eating patterns on MetS incidence need to be studied.

Primary eating factors affecting the MetS risk in different ethnicities are not evident. HEI includes a wide variation in magnitude of changes of diet-quality indicators. It can be used in the intervention of cardiometabolic risk conditions [8]. KHEI is a modified HEI for Korean eating habits to reflect Korean dietary patterns. KHEI can be used for monitoring dietary quality for adults. KHEI includes the adequacy domain of beneficial foods for health, moderation of sugar and sodium intake, and energy intake balance. The present study explored primary eating factors for MetS risk using KHEI. Total KHEI scores significantly lowered the MetS risk by about 0.5-fold in both genders, although individual items had different impacts on MetS in men and women. In men, fruit intake scores were significantly higher, while having breakfast scores were higher in women in the non-MetS group than in the MetS group of the KNHANES VI 2013–2017. The scores of milk and milk products were greater in the Non-MetS than the MetS in both genders. Therefore, better eating patterns might decrease the MetS risk in both genders. As a result, KHEI can be used for MetS prevention and intervention.

HEI is primarily composed of items with the adequacy and moderation domains. Each item contains a critical component such as fruit, vegetables, milk, and grains in the adequacy domain and refined grain, saturated fat, sodium, and sweets intake in the moderation domain of HEI in the USA [23]. HEI is modified as an alternative to HEI, including alcohol and multivitamin intake [24]. It has been reported that eating patterns can influence the MetS risk in different ethnicities. Different countries have developed HEI to evaluate the influence of eating habits on the risk of metabolic diseases. In Korea, having breakfast was included in the adequacy part in KHEI. Balanced eating scores were also added in KHEI [2]. The relationship between skipping breakfast and metabolic diseases remains a controversial issue, particularly in adults. Previous studies have demonstrated a positive association of not having breakfast with cardiometabolic risk factors, including obesity, hypertension, dyslipidemia, and hyperglycemia resulting from the metabolic syndrome in different countries, including Japan, Iran, and German, with different dietary patterns [25,26,27,28]. Inconsistent results might be related to no consideration of gender differences in adults. The present study demonstrated that women with MetS had lower scores of having breakfast (*p* = 0.025) after adjusting for all potential covariates. However, this was not shown in men. Breakfast consumption is significantly associated with lower BMI, body fat, insulin, homeostatic model assessment of insulin resistance (HOMA-IR), and MetS score, showing some gender differences [25]. However, consuming breakfast appears to improve glucose and insulin responses throughout the day compared to skipping breakfast based on some previous studies [29]. However, breakfast quality needs to be considered to prevent MetS. High carbohydrates in breakfast may elevate insulin resistance. Consumption of fermentable and viscous dietary fibers at breakfast can lower serum glucose and insulin concentrations, enhancing insulin sensitivity later in the day [29]. Gender differences and breakfast quality need to be considered.

In KHEI, scores of fruit and fruit juice intake were significant only in men, whereas vitamin C intake calculated from 24 h recall data was significantly higher in the Non-MetS group than in the MetS group in both genders in the present study. Dietary fiber intake was about 20–21 g/day in both MetS and Non-MetS groups of both genders. Differences in dietary fiber intake did not influence MetS incidence in the present study. However, the fruit intake score was significantly lower in the MetS group than in the Non-MetS group for men, while vitamin C intake was significantly higher in the Non-MetS group than in the MetS group in both genders. This suggested that fruit intake reduced MetS risk by acting as a vitamin C source and dietary fiber. A meta-analysis has demonstrated that fruit consumption (0.86 (0.77, 0.96)) and vegetable consumption (0.86 (0.80, 0.92)) have an inverse association with MetS risk in Asians [30]. A few studies have considered gender differences in vitamin C and dietary fiber intake for MetS risk [12,24]. MetS is associated with oxidative stress and chronic low-grade inflammation [18,31]. Poor vitamin C status can promote inflammation known to contribute to metabolic dysfunction, disturbing vitamin E trafficking through the gut-liver axis [32]. A higher intake of vitamin C is a major preventive nutrient for MetS, as shown in the present study and previous studies [18,33]. Unlike dietary fiber intake, a higher intake of vitamin C may lower the MetS risk, although its intake is higher than the recommended intake. Furthermore, 12-week supplementation of vitamin C (500 mg/day) resulted in a significant reduction in BMI, and a combination of physical activities and vitamin C supplements improved systolic blood pressure and dyslipidemia in metabolic syndrome patients [34]. Therefore, higher vitamin C intake above recommended levels might reduce the MetS risk.

Milk and milk products and calcium intake showed an inverse association with MetS risk in the present study. The relation between the calcium intake from milk and milk products and MetS has been controversial [35]. Calcium deficiency increases insulin resistance and visceral fat mass by down-regulating genes related to fat oxidation in estrogen-deficient animals [36], and it indicates the calcium deficiency is related to MetS risk. A meta-analysis has demonstrated that dietary calcium intakes are inversely related to MetS risk while a 300 mg increment of daily calcium intake can decrease the MetS risk by 7% (relative risk: 0.93; 95% CI: 0.87–0.99) [37]. Calcium and vitamin D interaction is also involved in MetS risk [38,39]. Since Asians have low serum 25-OH-vitamin D levels, higher C intake reduces the MetS risk [40]. Koreans have a low calcium intake (average of 450–530 mg/day), with women (about average 450 mg/day) having a lower calcium intake than men (average 520 mg/day). Calcium intake was significantly inversely associated with MetS only in women, although the intakes of milk and milk products had an inverse association with MetS risk in both genders. Therefore, sufficient milk and milk product intake might be a critical factor to alleviate MetS.

This study had the strength that healthy eating habits, especially having breakfast, fruit, and milk intake, might be critical for lowering the MetS risk in a large population study conducted in a complex, stratified, multistage probability cluster survey design. However, this study also had some limitations. First, the results cannot be interpreted as the cause-and-effect due to a cross-sectional study. Second, some foods were not included in the SQFFQ with 116 commonly consumed foods, leading to underestimating food intake. Third, a 1-day 24 h dietary recall might not reflect the usual daily nutrient intake, although a skilled technician conducted it to reduce measurement errors.

## 5. Conclusions

Overall scores of KHEI generated by KCDC showed an inverse association by 0.98-fold with MetS risk in both genders after adjusting for related covariates. Among adequacy intake in KHEI, having breakfast and fruit intake scores were inversely related to MetS risk only in men, while milk and milk product intake scores had the same trend of relationship with MetS risk in both genders. Calcium and vitamin C intake had an inverse association with MetS risk. Those with higher education and income showed less incidence of MetS. This finding was related to their higher KHEI scores. Therefore, the potential impact of eating habits and nutritional intake on MetS existed in both genders in Koreans. A daily dose of vitamin C (130 mg/d for men and 110 mg/d for women) and calcium (600 mg/d for both genders) may be linked to a lower MetS risk based on the present study. However, further investigation about the effect of vitamin C and calcium intake on MetS needs to be conducted in a large randomized clinical trial to demonstrate the cause-and-effect.

## Figures and Tables

**Table 1:** Distribution of socioeconomic and lifestyle variables according to metabolic syndrome.

Classification Variables		Metabolic Syndrome		
		Yes (*N* = 2128)	No (*N* = 10,189)	*p*-Value *
Sex	Female	1025 (19.7)	3861 (80.3)	<0.01
	Male	1103 (13.3)	6328 (86.7)	
Age group	20–29	96 (5.8)	1683 (94.2)	<0.01
	30–39	324 (12.3)	2536 (87.7)	
	40–49	512 (17.4)	2578 (82.6)	
	50–59	769 (24.5)	2382 (75.5)	
	60–64	427 (29.6)	1010 (70.4)	
Residence	Urban	1678 (15.6)	8567 (84.4)	<0.01
	Rural	450 (20.6)	1622 (79.4)	
Education	<High school	658 (29.3)	538 (70.7)	<0.01
	High school	679 (18.8)	2895 (81.2)	
	College	793 (11.9)	5756 (88.1)	
Income	1st Q	305 (24.6)	801 (75.4)	<0.01
	2nd Q	536 (17.1)	2435 (82.9)	
	3rd Q	659 (15.8)	3264 (84.2)	
	4th Q	619 (14.3)	3660 (85.7)	
Obesity	Lean	5 (0.7)	503 (99.3)	<0.01
	Normal	571 (6.5)	7248 (93.5)	
	Obese	1552 (37.6)	2438 (62.4)	
Smoking status	Current smoker	1130 (13)	6599 (87)	<0.01
	Ex-smoker	391 (18.4)	1665 (81.6)	
	Non-smoker	607 (23)	1925 (77)	
Drinking status	None	632 (20.8)	2355 (79.2)	<0.01
	Mild	957 (13)	5661 (87)	
	Moderate	218 (15.7)	1142 (84.3)	
	Severe	321 (23.9)	1031 (76.1)	
Exercise	Yes	973 (15.3)	5087 (84.7)	<0.01
	No	1155 (17.5)	5102 (82.5)	
Marriage	Yes	1916 (18.7)	8116 (81.3)	<0.01
	No	207 (9.1)	2072 (90.9)	
Survey year	2013	516 (15.1)	2745 (84.9)	<0.01
	2014	487 (15.5)	2493 (84.5)	
	2015	563 (17.5)	2388 (82.5)	
	2016	562 (17.6)	2563 (82.4)	

*: Chi-square test for each classification variable for metabolic syndrome.

**Table 2:** Distribution of socioeconomic and lifestyle variables according to Korean Healthy Eating Index (KHEI) score quartiles.

Classification Variables		KHEI Score				
		Q1 (*N* = 1177)	Q2 (*N* = 2341)	Q3 (*N* = 2379)	Q4 (*N* = 3120)	*p* Value *
Sex	Female	202 (6.1)	1168 (33.4)	1009 (27.2)	1214 (33.3)	<0.01
	Male	975 (19.6)	1173 (21.9)	1370 (24.6)	1906 (33.9)	
Age group	20–29	307 (18.7)	431 (29.4)	343 (21.9)	439 (30)	<0.01
	30–39	307 (14.7)	619 (31)	572 (26.8)	597 (27.5)	
	40–49	253 (11.3)	563 (26.9)	574 (26.8)	764 (35.1)	
	50–59	201 (7.8)	506 (24)	614 (27.8)	919 (40.4)	
	60–64	109 (10.5)	222 (23.3)	276 (27.3)	401 (38.9)	
Residence	Urban	977 (13.2)	1901 (27.2)	1957 (25.5)	2637 (34.1)	<0.01
	Rural	200 (12.1)	440 (29.5)	422 (27.7)	483 (30.7)	
Education	<high school	247 (14.6)	437 (29.8)	420 (25.3)	499 (30.2)	<0.01
	High school	358 (14.4)	652 (27.0)	669 (26.7)	844 (31.9)	
	College	572 (12.6)	1252 (27.2)	1290 (25.6)	1777 (35.2)	
Income	1st Q	169 (20.6)	238 (31.2)	182 (22.3)	184 (26)	<0.01
	2nd Q	367 (16.7)	604 (30.4)	559 (25)	656 (27.9)	
	3rd Q	365 (13)	752 (26.9)	818 (27.8)	939 (32.2)	
	4th Q	273 (8.6)	738 (25.1)	812 (25.6)	1335 (40.7)	
Obesity	Lean	81 (22)	107 (25.9)	111 (23.7)	131 (28.4)	<0.01
	Normal	758 (13.3)	1433 (26)	1541 (25.8)	2068 (34.9)	
	Obese	338 (11)	801 (31)	727 (26.3)	921 (31.7)	
Smoking status	Current smoker	790 (14)	1260 (22.9)	1490 (25.5)	2171 (37.6)	<0.01
	Ex-smoker	137 (9.6)	380 (26.6)	425 (28.2)	555 (35.6)	
	Non-smoker	250 (13.2)	701 (39.9)	464 (25)	394 (22)	
Drinking status	None	263 (11.5)	501 (25.2)	602 (26.9)	814 (36.4)	<0.01
	Mild	605 (12.6)	1164 (24.9)	1275 (26.1)	1804 (36.4)	
	Moderate	148 (14.9)	309 (32.6)	254 (24.6)	278 (27.9)	
	Severe	161 (15.5)	367 (38.3)	248 (24.3)	224 (21.9)	
Exercise	Yes	531 (12.2)	1099 (26.6)	1156 (25.9)	1603 (35.2)	<0.01
	No	646 (13.8)	1242 (28.5)	1223 (25.9)	1517 (31.8)	
Marriage	Yes	837 (11.4)	1794 (26.3)	1937 (27.1)	2600 (35.2)	<0.01
	No	340 (17.4)	545 (30.7)	441 (22.7)	519 (29.1)	
Year	2013	279 (11)	602 (26.2)	651 (26)	907 (36.7)	<0.01
	2014	284 (12.9)	585 (27.9)	590 (26.6)	749 (32.7)	
	2015	295 (13.6)	560 (27.8)	578 (25.9)	757 (32.8)	
	2016	319 (14.7)	594 (28.4)	560 (24.9)	707 (31.9)	

* Chi-square test for each classification variable for KHEI score.

**Table 3:** Adjusted ^†^ means and 95% confidence intervals of Korean Healthy Eating Index (KHEI) scores according to metabolic syndrome status with genders.

Classification		Female			Male		
		Metabolic Syndrome	Normal	*p*-Value *	Metabolic Syndrome	Normal	*p*-Value *
Adequacy	Have breakfast	6.72 (6.44~6.99)	7.06 (6.95~7.18)	0.019	6.66 (6.38~6.94)	6.73 (6.59~6.87)	0.65
	Mixed grains intake	4.15 (4.02~4.27)	4.13 (4.07~4.18)	0.768	3.80 (3.65~3.96)	3.78 (3.71~3.86)	0.802
	Total fruits intake	3.72 (3.60~3.84)	3.78 (3.74~3.83)	0.319	2.27 (2.14~2.41)	2.52 (2.45~2.59)	0.002
	Fresh fruits intake	3.61 (3.49~3.73)	3.68 (3.63~3.73)	0.305	2.01 (1.88~2.14)	2.25 (2.18~2.32)	0.002
	Total vegetable intake	4.85 (4.81~4.89)	4.88 (4.86~4.89)	0.225	4.76 (4.70~4.82)	4.80 (4.77~4.83)	0.231
	Vegetable intake excluding kimchi and pickled vegetables	4.29 (4.19~4.39)	4.33 (4.29~4.36)	0.456	3.72 (3.60~3.83)	3.79 (3.74~3.85)	0.224
	Meat, fish, eggs, and beans intake	3.91 (3.76~4.06)	4.02 (3.97~4.07)	0.168	3.93 (3.81~4.06)	3.95 (3.89~4.00)	0.873
	Milk and milk products intake	3.23 (2.92~3.533)	3.75 (3.63~3.87)	0.002	3.05 (2.75~3.34)	3.44 (3.29~3.59)	0.026
Moderation	% of energy from saturated fatty acids	9.46 (9.39~9.54)	9.40 (9.36~9.43)	0.068	9.47 (9.39~9.55)	9.46 (9.42~9.49)	0.81
	Sodium intake	5.29 (5.04~5.54)	5.26 (5.15~5.36)	0.806	3.84 (3.57~4.12)	3.85 (3.72~3.98)	0.969
	% of energy from sweets and beverage	3.73 (3.58~3.88)	3.64 (3.57~3.70)	0.268	4.11 (3.97~4.26)	3.96 (3.89~4.03)	0.060
Balance of energy intake	% of energy from CHO	3.16 (3.03~3.29)	3.21 (3.16~3.26)	0.452	3.21 (3.06~3.36)	3.29 (3.22~3.35)	0.337
	% of energy from fat	4.01 (3.89~4.13)	4.14 (4.11~4.18)	0.044	3.98 (3.86~4.1)	4.02 (3.97~4.07)	0.546
	Energy intake	4.03 (3.91~4.16)	3.93 (3.88~3.98)	0.123	3.93 (3.78~4.08)	3.94 (3.88~4.01)	0.861
KHEI for all scores		63.13 (62.42~63.84)	64.21 (63.92~64.5)	0.005	57.83 (57.11~58.55)	58.91 (58.57~59.25)	0.007

^†^ adjusted by age, residence, region, education, obesity, income, drinking status, smoking status, marriage, and exercise. * *p*-value by Satterthwaite Chi-Square test.

**Table 4:** According to genders and metabolic syndrome, adjusted mean ^†^ and 95% confidence intervals of macronutrient intake were calculated by 24 h recall.

	Female			Male		
	Metabolic Syndrome	Normal	*p*-Value *	Metabolic Syndrome	Normal	*p*-Value *
Energy (kcal/d)	1808 (1764~1853)	1812 (1792~1832)	0.89	2401 (2339~2463)	2406 (2374~2438)	0.892
Fat (En%)	18.0 (17.6~18.4)	18.6 (18.5~18.8)	0.006	17.4 (17.0~17.8)	17.6 (17.4~17.8)	0.315
Protein (En%)	13.4 (13.2~13.5)	13.5 (13.5~13.6)	0.061	12.7 (12.5~12.8)	12.6 (12.6~12.7)	0.167
Carbohydrate (En%)	65.6 (65.1~66.2)	65.1 (64.9~65.3)	0.069	61.2 (60.6~61.8)	61.4 (61.2~61.7)	0.901
Fiber (g/1000 kcal)	8.89 (8.70~9.08)	8.91 (8.82~9.00)	0.271	11.17 (10.94~11.40)	11.31 (11.22~11.40)	0.842
Calcium (mg/1000 kcal)	213.3 (208.3~218.3)	218.2 (216.0~220.3)	0.002	248.7 (242.1~255)	259.8 (257.4~262.1)	0.09
Iron (mg/1000 kcal)	6.35 (6.24~6.45)	6.31 (6.26~6.35)	0.464	7.235 (7.121~7.348)	7.281 (7.238~7.325)	0.492
V-C (mg/1000 kcal)	40.4 (38.6~42.2)	45.0 (43.9~46.2)	0.003	65.2 (62.3~68.2)	70.0 (68.8~71.2)	0.001

^†^ adjusted by age, residence, region, education, obesity, income, drinking status, smoking status, marriage, and exercise. * *p*-value by Satterthwaite chi-square.

**Table 5:** Odds ratios (95% confidence interval (CI)) for having metabolic syndrome by Korean Healthy Eating Index (KHEI) after adjustments for covariates.

	KHEI	Female	Male	All
Model 1	Q1	Reference (1.000)	Reference (1.000)	Reference (1.000)
	Q2	0.774 (0.606~0.988)	0.694 (0.486~0.99)	0.773 (0.639~0.935)
	Q3	0.697 (0.544~0.893)	0.639 (0.451~0.905)	0.694 (0.57~0.844)
	Q4	0.526 (0.42~0.657)	0.475 (0.334~0.677)	0.528 (0.439~0.636)
Model 2	Q1	Reference (1.000)	Reference (1.000)	Reference (1.000)
	Q2	0.818 (0.628~1.064)	0.556 (0.379~0.816)	0.75 (0.61~0.923)
	Q3	0.885 (0.678~1.155)	0.527 (0.362~0.767)	0.743 (0.602~0.916)
	Q4	0.692 (0.542~0.884)	0.4 (0.272~0.587)	0.582 (0.475~0.713)
Model 3	Q1	Reference (1.000)	Reference (1.000)	Reference (1.000)
	Q2	0.822 (0.631~1.071)	0.593 (0.399~0.882)	0.765 (0.619~0.946)
	Q3	0.892 (0.682~1.166)	0.609 (0.412~0.902)	0.794 (0.64~0.986)
	Q4	0.702 (0.55~0.897)	0.486 (0.323~0.73)	0.644 (0.52~0.798)

Model 1; adjusted for sex, age, residence, and region. Model 2; adjusted for model 1 + education, income, marriage, and obesity. Model 3: adjusted for model 2+ smoking, alcohol, regular exercise.

## Data Availability

The data presented in this study are available on request from the corresponding author.

## Supplementary Materials

The following are available online at https://www.mdpi.com/article/10.3390/nu13041312/s1, Table S1: Components of and scoring standards for Korean Healthy Eating Index.
