# Supplementary material for: A Healthy Diet Rich in Calcium and Vitamin C Is Inversely Associated with Metabolic Syndrome Risk in Korean Adults from the KNHANES 2013–2017
Source: Nutrients. 2021 Apr 16;13(4):1312. doi: 10.3390/nu13041312 (PMC8073625; doi:10.3390/nu13041312)
Supplement: Supplementary file 1 [file nutrients-13-01312-s001.zip › nutrients-1158319-supplementary.pdf]

Supplementary Table S1. Components of and scoring standards for Korean Healthy Eating Index

Components (score range)	Reference for score of each component	
	Maximum score	Minimum score
Having breakfast (0–10)	5–7 day/week	0 day/week
Whole grain (0–5)	≥0.3 serving/day	0 serving/day
Total fruit, including juice (0–5)	Men: (aged 19–64) ≥3 serving/day, (aged≥65) ≥2 serving/day<br>Women: (aged 19–64) ≥2 serving/day, (aged≥65) ≥1 serving/day	0 serving/day
Fruit, excluding juice (0–5)	Men: (aged 19–64) ≥1.5 serving/day, (aged≥65) ≥1 serving/day<br>Women: (aged 19–64) ≥1 serving/day, (aged≥65) ≥0.5 serving/day	0 serving/day
Total vegetable, including Kimchi and pickles (0–5)	Men: ≥8 serving/day<br>Women: (aged 19–64) ≥8 serving/day, (aged≥65) ≥6 serving/day	0 serving/day
Vegetable, excluding Kimchi and pickles (0–5)	Men: ≥5 serving/day<br>Women: (aged 19–64) ≥5 serving/day, (aged≥65) ≥3 serving/day	0 serving/day
Meat, fish, eggs, and legumes (0–10)	Men: (aged 19–64) ≥5 serving/day, (aged≥65): ≥4 serving/day<br>Women: (aged 19–64) ≥4 serving/day, (aged≥65) ≥2.5 serving/day	0 serving/day
Milk and dairy (0–10)	≥1 serving/day	0 serving/day
Sodium (0–10)	≤2,000 mg/day	>6,500 mg/day
Saturated fatty acid (0–10)	≤7% of energy	>10 % of energy
Empty calorie foods (0–10)	≤10% of energy	>20 % of energy
Carbohydrate (0–5)	55–65% of energy	<50%, >75% of energy
Fat (0–5)	15–30% of energy	<10%, >35% of energy
Total energy (0–5)	75–125% of EER by sex and age group	<60%, >140% of EAR

EER = estimated energy requirement.
